# SARS-CoV-2 expresses a microRNA-like small RNA able to selectively repress host genes

**DOI:** 10.1073/pnas.2116668118

**Published:** 2021-12-13

**Authors:** Paulina Pawlica, Therese A. Yario, Sylvia White, Jianhui Wang, Walter N. Moss, Pei Hui, Joseph M. Vinetz, Joan A. Steitz

**Affiliations:** ^a^Department of Molecular Biophysics and Biochemistry, Yale University School of Medicine, New Haven, CT 06536;; ^b^HHMI, Yale University School of Medicine, New Haven, CT 06536;; ^c^Department of Pathology, Yale University School of Medicine, New Haven, CT 06536;; ^d^Roy J. Carver Department of Biochemistry, Biophysics, and Molecular Biology, Iowa State University, Ames, IA 50011;; ^e^Section of Infectious Diseases, Department of Internal Medicine, Yale University School of Medicine, New Haven, CT 06520

**Keywords:** micoRNA, SARS-CoV-2, noncoding RNA

## Abstract

Severe acute respiratory syndrome coronavirus 2 (SARS-CoV-2), the causative agent of coronavirus disease (COVID-19), continues to be a pressing health concern. In this study, we investigated the impact of SARS-CoV-2 infection on host microRNA (miRNA) populations in three human lung-derived cell lines, as well as in nasopharyngeal swabs from SARS-CoV-2–infected individuals. We did not detect any major and consistent differences in host miRNA levels after SARS-CoV-2 infection. However, we unexpectedly discovered a viral miRNA-like small RNA, named CoV2-miR-O7a (for SARS-CoV-2 miRNA-like ORF7a-derived small RNA). Its abundance ranges from low to moderate as compared to host miRNAs and it associates with Argonaute proteins—core components of the RNA interference pathway. We identify putative targets for CoV2-miR-O7a, including Basic Leucine Zipper ATF-Like Transcription Factor 2 (BATF2), which participates in interferon signaling. We demonstrate that CoV2-miR-O7a production relies on cellular machinery, yet is independent of Drosha protein, and is enhanced by the presence of a strong and evolutionarily conserved hairpin formed within the ORF7a sequence.

## Significance

We discovered that severe acute respiratory syndrome coronavirus 2 (SARS-CoV-2) expresses a small viral noncoding RNA, named CoV2-miR-O7a (for SARS-CoV-2 miRNA-like ORF7a-derived small RNA). CoV2-miR-O7a associates with the cellular RNA interference machinery and has the potential to regulate host transcripts, likely via target slicing. The production of CoV2-miR-O7a relies on cellular machinery and the formation of a strong hairpin within ORF7a sequences. This newly described CoV2-miR-O7a may contribute to SARS-CoV-2 pathogenesis and could become a target for therapeutic intervention.

Coronaviruses are large single-stranded positive-sense RNA viruses that infect various animals; in humans coronaviral infection results in mild to severe respiratory disease. β-coronaviruses, such as severe acute respiratory syndrome coronavirus (SARS-CoV) and Middle East respiratory syndrome coronavirus (MERS-CoV), cause very severe disease in humans, while the recently emerged severe acute respiratory syndrome coronavirus 2 (SARS-CoV-2) results in coronavirus disease (COVID-19) characterized by wide array of symptoms from mild to serious illness. SARS-CoV-2 is the cause of the COVID-19 pandemic due to its high transmissibility and emergence of novel variants (1). It is crucial to understand all aspects of SARS-CoV-2 pathogenesis in order to develop multiple complementary tools to combat the constantly adapting virus.

microRNAs (miRNAs) are ∼22-nucleotide (nt)-long small noncoding RNAs (ncRNAs) involved in posttranscriptional regulation of gene expression in animals and plants (2). During canonical miRNA biogenesis, RNA polymerase II transcribes primary miRNAs (pri-miRNAs) characterized by formation of strong hairpins that are recognized and cleaved by the endonuclease Drosha, resulting in ∼70-nt-long precursor miRNAs (pre-miRNAs). Pre-miRNAs are exported to the cytoplasm, where they are subjected to yet another cleavage by Dicer, giving rise to ∼22-nt-long miRNA duplexes, which are then loaded onto Argonaute (Ago) proteins. The passenger strand is removed, and the mature miRNA guides Ago proteins to partially complementary target sequences, usually located in the 3′ untranslated region (UTR) of messenger RNAs (mRNAs). miRNA binding leads to translation inhibition and/or mRNA decay by decapping and deadenylation. In the special case where the miRNA is perfectly complementary to its target mRNA, it directs Ago2 (one of four Ago proteins in humans) to cleave the mRNA target, a function typically associated with small interfering RNAs (siRNAs) (2). Both of these modes of target repression belong to the RNA interference (RNAi) pathway, but Ago2-mediated cleavage is more potent in target silencing and requires lower intracellular copy numbers than the canonical miRNA action mode (3). Importantly, miRNA profiles differ between cell types and developmental stages, and a single miRNA can down-regulate multiple transcripts, precisely regulating gene expression dependent on the cell’s needs. In humans, most mRNAs are regulated by miRNAs, and aberrant miRNA levels are linked to disease (2).

Viruses often hijack the miRNA pathway, either by depleting host miRNAs or by producing their own miRNAs (reviewed in refs. 4 and 5). Some examples of host miRNA regulation include selective host miRNA decay mediated by certain herpesviral transcripts in a process called target-directed miRNA degradation (reviewed in refs. 6 and 7) and widespread miRNA polyadenylation by poxvirus poly(A) polymerase, which results in miRNA decay (8). Viruses can produce their own miRNAs, often via noncanonical biogenesis pathways; cytoplasmic viruses especially have devised multiple strategies to bypass the requirement for nuclear Drosha (4, 9). It has been proposed that coronaviruses, SARS-CoV and SARS-CoV-2, produce small viral RNAs (svRNAs) involved in viral pathogenesis independent of the RNAi pathway (10, 11). Recent studies suggested the existence of miRNA-like RNAs produced by SARS-CoV-2, which might regulate inflammation and interferon (IFN) signaling (12, 13).

In this study, by using a small RNA-sequencing (smRNA-seq) library preparation protocol that selectively enriches for miRNAs, we unexpectedly discovered an miRNA-like viral small RNA, named CoV2-miR-O7a, expressed by SARS-CoV-2. We show that it associates with Ago proteins and has the potential to regulate host transcripts, likely via target slicing. We demonstrate that CoV2-miR-O7a production relies on cellular machinery but is independent of Drosha proteins and is enhanced by the presence of a strong hairpin formed within ORF7a sequences. This newly described viral ncRNA may contribute to SARS-CoV-2 pathogenesis and could become a target for therapeutic intervention.

## Results

### SARS-CoV-2 Infection Has Minimal Impact on the miRNA Population of Its Host Cell.

**Fig. 1.**
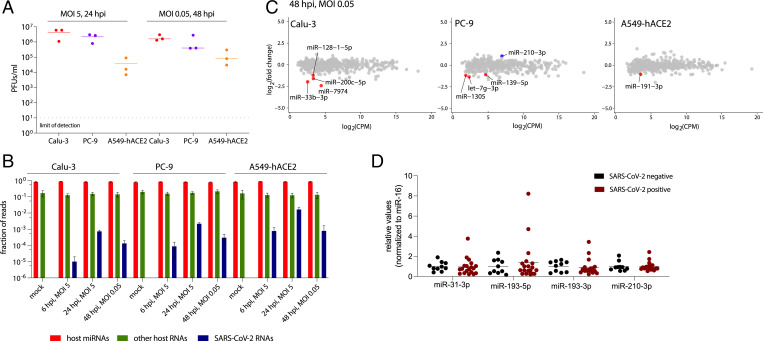
SARS-CoV-2 infection has minimal impact on host miRNA levels. (*A*) SARS-CoV-2 replicates in the three human lung cell lines used in this study, as demonstrated by plaque assays. (*B*) Small RNA library preparation strongly enriches host miRNAs. A bar graph summarizing the origin of obtained reads across the sequenced conditions. Error bars signify SD. (*C*) Plots summarizing the fold change in host miRNA levels 48 hours postinfection (hpi) with SARS-CoV-2, *n* = 3. Colored dots denote miRNAs that significantly (*P* < 0.05) changed (at least twofold). Significance was calculated with edgeR. (*D*) The relative levels of selected miRNAs are unchanged in nasopharyngeal samples from SARS-CoV-2–infected individuals versus uninfected controls. PFUs, plaque-forming units.

To explore how SARS-CoV-2 affects host miRNA populations, we infected three lung-derived human cell lines either at high multiplicity of infection (MOI of 5) to detect the possible direct impact of the incoming virions on host miRNAs or at low MOI (0.05) to assess the effect of viral replication on these small RNA species. We used non-small-cell lung cancer cells Calu-3, lung adenocarcinoma cells A549 transduced with the human viral receptor, angiotensin-converting enzyme 2 (ACE2), and lung adenocarcinoma cells PC-9, all of which we found to support SARS-CoV-2 replication (red, yellow, and blue dots, respectively, in Fig. 1*A*). SARS-CoV-2 replicated better in Calu-3 and PC-9 cell lines than in the A549-hACE2 cell line, possibly because the viral receptor is greatly overexpressed in the last cell line (*SI Appendix*, Fig. S1*A*), which could sequester budding virions at the cell surface.

Small RNA sequencing was performed at both 6 h and 24 h postinfection (hpi) at high MOI and 48 hpi at low MOI, as well as for the uninfected controls (at the 24-h time point). The vast majority (84% ± 5%) of obtained reads mapped to host miRNAs (red bars in Fig. 1*B*) with an average read length of 22 or 23 nt (*SI Appendix*, Fig. S1*B*). Overall, only a small fraction of small RNA reads mapped to the viral genome (0.01 to 1.63% depending on conditions; blue bars in Fig. 1*B*). This number is in agreement with similar studies of small viral RNAs in other RNA viruses (10–12).

SARS-CoV-2 infection resulted in minimal alteration of some host miRNA levels, but no miRNA displayed a uniformly significant change across all three cell lines (red and blue dots in Fig. 1*C* and *SI Appendix*, Fig. S1*C*). To identify any consistent shifts in host miRNA levels across the three cell lines, we generated a heat map of fold changes for miRNAs that were significantly altered, by at least twofold, in at least one condition (*SI Appendix*, Fig. S1*D*). Some of these trends were further examined by Northern blotting of RNA from Calu-3 cells infected with SARS-CoV-2 at low MOI. The most reproducibly changed (up-regulated) miRNA, although not reaching statistical significance, was miR-210-3p (*SI Appendix*, Fig. S1*E*). Interestingly, Gene Ontology term analyses for validated miR-210-3p targets (14) revealed that this miRNA is involved in regulating responses to cellular stimuli, such as hypoxia (*SI Appendix*, Fig. S1*F*). In addition, in two datasets obtained from lung biopsies of COVID-19 patients (15, 16) miR-210-3p targets were down-regulated to a greater extent than other human mRNAs (*SI Appendix*, Fig. S1*G*).

Of note, the changes in miRNA levels observed by smRNA-seq were not detected by TaqMan RT-qPCR from nasopharyngeal swabs from SARS-CoV-2–positive individuals (Fig. 1*D*). Interestingly, analysis of unnormalized Ct values revealed that after SARS-CoV-2 infection the overall miRNA abundance increased, while the levels of ncRNAs from two other small RNA classes (small nuclear RNA U6B and small nucleolar RNA U44) decreased (*SI Appendix*, Fig. S1*H*), suggesting that miRNAs might escape the viral host shut-off effect (17) . This agrees with the notion that miRNAs can be very stable, e.g., in circulating exosomes (18, 19). Thus, it may be possible to devise small RNA-mediated treatments to treat COVID-19. Overall, we did not find any major impact of SARS-CoV-2 infection on host-cell miRNA populations.

### SARS-CoV-2 Expresses an miRNA-Like Small RNA Derived from the ORF7a Sequence.

**Fig. 2.**
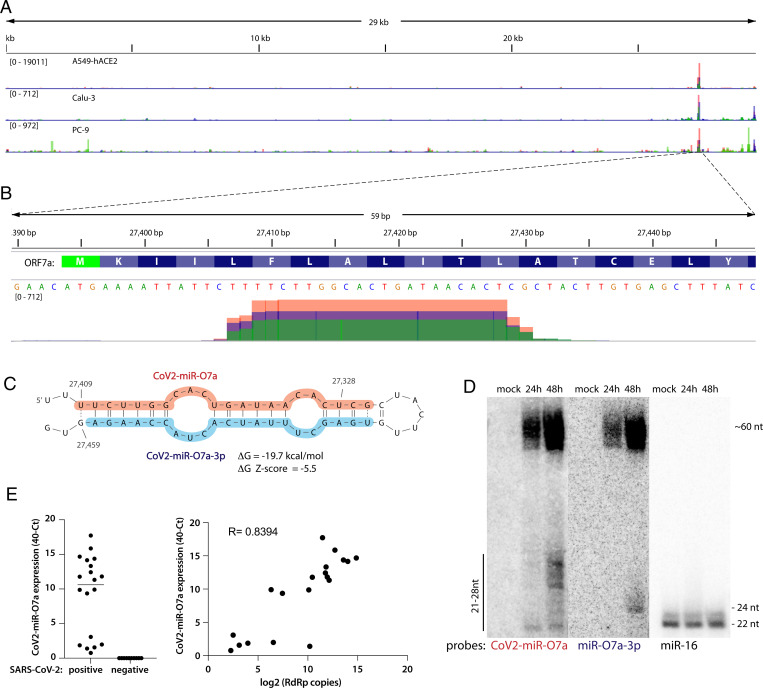
SARS-CoV-2 expresses a small RNA derived from the ORF7a sequence. (*A*) Viral small RNA reads obtained from the three cell lines infected with SARS-CoV-2 map to a single distinct peak within the viral genome (data for MOI 5, 24 hpi are shown). The replicates for each cell line were overlayed on a single track (represented by different colors) and are normalized to 10^7^ total reads. (*B*) The reads coming from SARS-CoV-2 (∼20 to 25 nt long) map near the beginning of the ORF7a gene (encoded amino acids are shown above the nucleotide sequence). Data from Calu-3 cells are shown. (*C*) CoV2-miR-O7a forms a hairpin with the sequence immediately downstream in the ORF7a transcript. Shaded nucleotides indicate the sequences detected by Northern blot probes: orange for 5p and blue for 3p. (*D*) CoV2-miR-O7a and its precursor can be detected by Northern blotting of extracts from Calu-3 cells infected with SARS-CoV-2 at MOI 0.05. (*E*) As measured by custom TaqMan RT-qPCR, CoV2-miR-O7a is present in nasopharyngeal samples from SARS-CoV-2–infected individuals (*Right*), with its levels correlating with viral load (*Left*).

Of the reads derived from SARS-CoV-2, 5.2% ± 2.8% mapped to a single peak within the ORF7a sequence (Fig. 2*A* and *SI Appendix*, Fig. S2*A*). ORF7a encodes a SARS-CoV-2 accessory protein, a putative type-I transmembrane protein involved in antagonizing the type I IFN response (reviewed in ref. 20). The reads mapping to the ORF7a sequence are ∼20 to 25 nt long (Fig. 2*B*). Because the small RNA libraries were dominated by host miRNAs and a U [the preferred 5′-terminal nucleotide for Ago loading ] is present at the 5′ end of this RNA, we interrogated whether it could represent a viral miRNA-like small RNA.

Since miRNA biogenesis relies on formation of RNA hairpins, we asked whether such an RNA structure could be found within the viral ORF7a sequence. Indeed, the putative viral miRNA together with its downstream region forms a strong hairpin (Fig. 2*C*) whose folding energy is unusually low given the AU-rich nucleotide composition (ΔG = −19.7 kcal/mol), suggesting that it arose through an evolutionarily driven process. Indeed, the thermodynamic Z-score is −5.5 (meaning that the wild-type sequence is more than 5 SDs more stable than random). In addition, a comprehensive analysis of RNA structural motifs in SARS-CoV-2 found that this hairpin is one of the few (nine) that show evidence of statistically significant sequence covariation—a feature of evolutionary conservation (22). The formation of the hairpin is also in agreement with previously reported structure probing reactivity datasets (23–26), where the only reactive nucleotides occur in loop regions. Since the newly identified small RNA is derived from this hairpin—which is a hallmark of miRNA biogenesis (27)—we named it CoV2-miR-O7a (for SARS-CoV-2 miRNA-like ORF7a-derived small RNA). In addition, there exists sequence and structure similarity of the RNA hairpin between SARS-CoV-2, bat coronavirus RaTG13, and a pangolin coronavirus (*SI Appendix*, Fig. S2*B*).

CoV2-miR-O7a and its pre-miRNA-sized precursor are detectable by Northern blotting of RNA from Calu-3 cells infected with SARS-CoV-2 at low MOI (Fig. 2*D*). Interestingly, the most prevalent species of CoV2-miR-O7a observed by Northern blotting are 25 to 27 nt long, longer than those identified by small RNA sequencing. Since the small RNA library was size-selected, this could mean that CoV2-miR-O7a is in fact more abundant than when quantified by smRNA-seq. However, because miRNAs are usually ∼22 nt long, CoV2-miR-O7a or its precursor could have an additional RNAi-independent function. Importantly, CoV2-miR-O7a was detected in individuals infected with SARS-CoV-2 (Fig. 2*E*) and its abundance correlated positively with viral load/genomic RNA (as measured by the levels of RNA-dependent RNA polymerase, RdRp).

### CoV2-miR-O7a Binds Ago and Can Repress Human mRNAs.

**Fig. 3.**
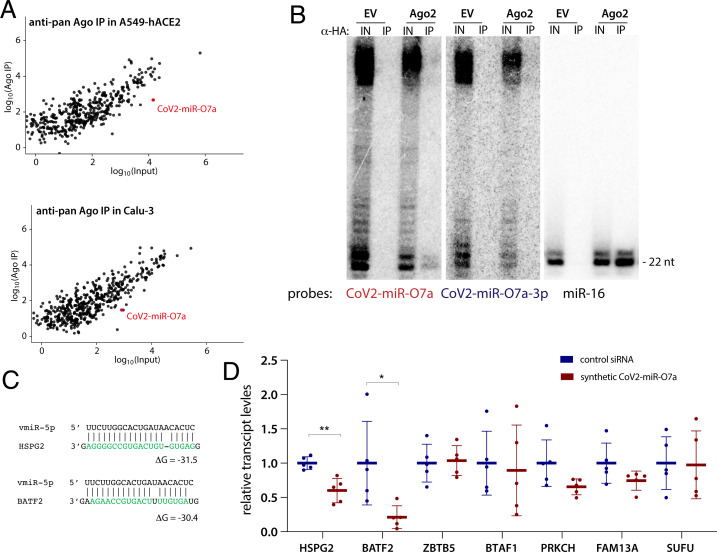
CoV2-miR-O7a associates with Argonaute and has the capacity to repress the levels of host transcripts. (*A*) CoV2-miR-O7a associates with Ago proteins and its levels are comparable to those of moderately expressed host miRNAs. Anti-pan-Ago RNA immunoprecipitation (IP) followed by sequencing was performed on extracts from Calu-3 and A549-hACE2 cells infected with SARS-CoV-2 at MOI 5 for 24 h. Each plot shows the average of three independent experiments. (*B*) CoV2-miR-O7a is selectively loaded on Ago2. A representative Northern blot for CoV2-miR-O7a and CoV2-miR-O7a-3p showing anti-HA IP from Calu-3 cells transduced with either empty vector (EV) or with FLAG-HA–tagged Ago2 (Ago2). IN, input, 10%. The miR-16 lanes provide size markers. (*C*) Predicted interactions between CoV2-miR-O7a and sequences from the coding sequences of two targeted host mRNAs. (*D*) Synthetic CoV2-miR-O7a down-regulates host gene expression. HEK293T cells were transfected with synthetic CoV2-miR-O7a (annealed to a passenger strand) and levels of seven selected host transcripts shown in Table 1 were measured by RT-qPCR 24 h later. Means with SD are shown. *P < 0.05, **P < 0.01 as calculated by two-tailed paired *t* test.

Since the miRNA program is executed by Argonaute proteins, we asked whether these host proteins bind CoV2-miR-O7a. We performed anti-pan-Ago RNA immunoprecipitation (RIP) on extracts from Calu-3 and A549-hACE2 cells infected with SARS-CoV-2 at high MOI, followed by small RNA sequencing. In both cell lines, CoV2-miR-O7a associates with Ago proteins, although to a lesser extent than most host-cell miRNAs; its expression levels are comparable to low or moderately expressed host miRNAs (Fig. 3*A*). To obtain enough material for Northern blotting experiments, we performed an anti-hemagglutinin (HA) RIP from Calu-3 cells transduced with either empty vector or FLAG-HA–tagged human Ago2 . Although weak, Ago2 selectively binds CoV2-miR-O7a, but not CoV2-miR-O7a-3p. In these experiments, we observed slight RNA degradation likely arising during the SARS-CoV-2 inactivation step (30-min incubation at room temperature). Perhaps this is the reason why CoV2-miR-O7a bands in Fig. 3*B* migrate somewhat faster than those in Fig. 2*D*.

The role of Ago-associated small RNAs is to repress targeted mRNAs via sequence complementarity. We thus computationally searched the repository of human mRNA transcripts (obtained from GENECODE) for putative CoV2-miR-O7a targets. Considering the moderate expression levels of CoV2-miR-O7a as compared to those of host miRNAs, we hypothesized that CoV2-miR-O7a might rely on Ago2’s ability to cleave its mRNA target. Since Ago2-mediated target cleavage tolerates a limited number of mismatches (29, 30) , we searched for mRNAs with high complementarity to CoV2-miR-O7a (Table 1 and Fig. 3*C*) and selected some of these for further validation.

**Table 1.** Identification of human transcripts highly complementary to CoV2-miR-O7a

Gene	Sequence	Seed base pairing	Gene	CoV2-miR-O7a	ΔG	Location
HSPG2	GGAGTGTGTCAGTGCCGGGGAG	Yes	.((((((((((((((((((((.	))))))))))))))).))))).	−31.5	CDS
SUFU	TGAGTGGTGTCAGTGCCAAGT	No	.((((((..((((((((((((.	..))))))))))))..))))))	−31.1	CDS
BATF2	CCCTAGTAGTGTTTTCAGTGCCAAGAAG	Yes	.((((((.(((((((((((((.	))))))))))))).)))))).	−30.4	CDS
ZBTB5	GGAGTTGTGGTCAGTGCCGAGAAG	Yes	.(((.(((.((((((((((((((.	)))))))))))))).)))))).	−29.9	CDS
SLC30A3	CGGGATACGCTGTTGTCGGTGCCAGGAGT	Yes	.((((......(((((((((((((((((.	.)))))))))))))))))))))	−28.9	CDS
LAMA3	GGAGTGTGCCAGTGCCGAGAG	Yes	.((((((..(((((((((((.	.)))))))))))...)))))).	−28.7	CDS
MCF2L2	GGGGTGTTCGTGTTAGTGCCAAGAT	Yes	.((((((...((((((((((((((.	.)))))))))))))))))))).	−28.5	3′UTR
BTAF1	TGAGTGGAATCCAGTGCCGAGAAC	Yes	.((((((..((.((((((((((((.	))))))))))))))..))))))	−28.4	CDS
BUD23	TGGTGGGATCAGTGCCAGGCA	No	.((((..((((((((((((.	.))))))))))))..)))).	−28.3	3′UTR
FAM120C	CCGAGTGGGGCCGTCGGTGCCAGGCC	No	.((((((.....((((((((((((.	.))))))))))))..))))))	−28.3	CDS
PRKCH	GGAGTGTTTGGGAAACAGGGTTATCAGTGCCAAGT	No	.((((((..............(((((((((((((.	.))))))))))))))))))).	−28.3	CDS
FAM13A	AGGGTGCTATTAGTGCCAAGTT	No	.(((((.(((((((((((((.	.))))))))))))).))))).	−28	CDS

CDS, coding DNA sequence. UTR, untranslated region.

Due to the widespread host-shutoff effect after infection with SARS-CoV-2 (17), we were unable to perform meaningful quantification of mRNAs from infected cells. We thus transfected synthetic CoV2-miR-O7a (annealed to a passenger strand) into HEK293T cells and assayed transcript levels of predicted target mRNAs (Fig. 3*C*). Two mRNAs, Basic Leucine Zipper ATF-Like Transcription Factor 2 (BATF2) and Heparan Sulfate Proteoglycan 2 (HSPG2), were significantly down-regulated (Fig. 3*D*). BATF2 has been linked to INF-gamma signaling via association with Irf1 (31). HSPG2 is the core protein of a large multidomain proteoglycan that binds and cross-links many extracellular matrix components; interestingly, it participates in the attachment of some viruses, including the coronavirus NL63 (32). We hypothesize that CoV2-miR-O7a has the potential to repress human mRNAs to evade the host IFN response and perhaps to inhibit viral superinfection.

### Production of Functional CoV2-miR-O7a Depends on Cellular Machinery.

**Fig. 4.**
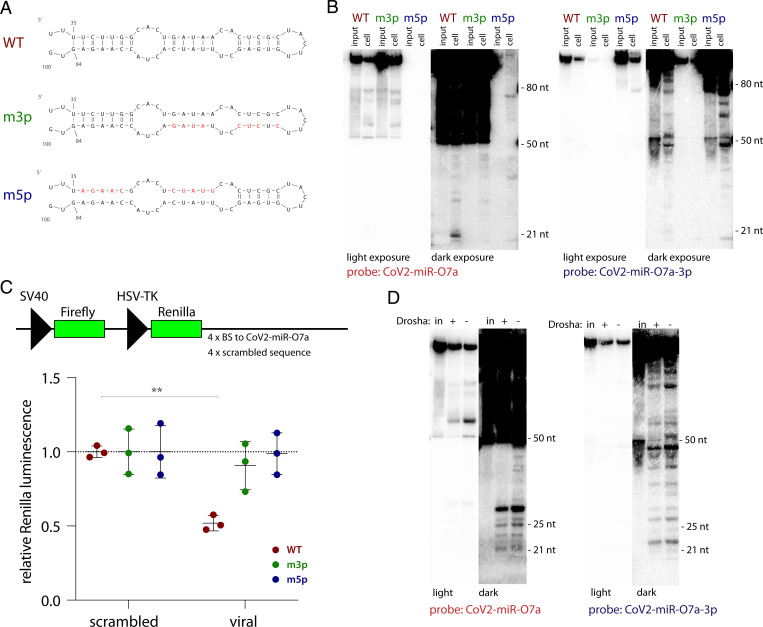
Production of functional CoV2-miR-O7a is Drosha-independent. (*A*) In vitro transcripts used in this study. Red nucleotides represent introduced mutations. (*B*) CoV2-miR-O7a processing inside cells is enhanced by the formation of a hairpin involving the downstream sequence. A representative Northern blot of RNAs isolated from HEK293T cell 6 h posttransfection with each of the in vitro transcribed 100-nt-long RNA sequences shown in *A*. Input, 10%. (*C*) CoV2-miR-O7a processed inside HEK293T cells is capable of repressing a reporter. (*Top*) Schematic of the psiCHECK-based luciferase reporters used. Each of the four binding sites (BS) was 21 nt long, separated by unique 20-nt-long spacers. (*Bottom*) CoV2-miR-O7a generated from an in vitro-transcribed hairpin can regulate a luciferase reporter gene containing downstream complementary sites in HEK293T cells. Means with SD are shown. **P < 0.01 as calculated by two-tailed paired *t* test. (*D*) CoV2-miR-O7a processing is Drosha-independent. A representative Northern blot of RNAs isolated from either Huh7.5 or Huh7.5 Drosha knockout cells (33) 6 h posttransfection with in vitro-transcribed 100-nt-long viral RNA sequence. In, input, 10%.

To assess whether CoV2-miR-O7a biogenesis can occur in a virus-free system, we transfected in vitro-transcribed RNA into HEK293T cells. A 100-nt-long viral RNA sequence (WT), alongside with its counterparts containing mutations either in the 3′ portion (m3p) or in the 5′ portion (m5p) of the hairpin (Fig. 4*A*), were transfected into cells and RNA samples collected after 6 h were analyzed by Northern blotting (Fig. 4*B*). The WT sequence was processed inside cells to produce a 21-nt-long band of CoV2-miR-O7a, while the m3p did not yield efficient production of an RNA of such size.

To investigate whether the produced RNA functionally associates with Ago proteins, we transfected in vitro-transcribed RNA together with luciferase reporters containing 3′ UTR sequences complementary to either CoV2-miR-O7a or scrambled controls. The luciferase activity of the reporter containing viral sequences was significantly decreased in the presence of the WT ORF7a RNA (Fig. 4*C*) but not by the other constructs. Finally, we used Drosha knockout cell lines (33) to show that the production of CoV2-miR-O7a occurs independently of this enzyme’s activity (Fig. 4*D*). These results clearly demonstrate that CoV2-miR-O7a is processed from a hairpin independently of viral proteins and of cellular Drosha, and that it can be functionally loaded on Ago.

## Discussion

Here, we began by investigating the impact of SARS-CoV-2 infection on host miRNA populations using three human lung-derived cell lines (at various MOIs) and found no consistent changes detected in all three cell lines (Fig. 1*C* and *SI Appendix*, Fig. S1*C*). We also assessed the levels of selected miRNAs in patient samples and again did not detect major differences after SARS-CoV-2 infection (Fig. 1*D*). Other groups have also ventured into examining miRNA profiles during SARS-CoV-2 infection, but there is little overlap between these prior studies (34–36) and our results. Differences between the outcomes likely stem from the use of different methods of smRNA-seq library preparation (37, 38). Our libraries were prepared with the NEXTflex v2 kit, which has been shown to be highly selective for miRNAs (37), and contained ∼84% host miRNAs (Fig. 1*B*), while libraries that rely on template switching used by others (34) yield only ∼17% miRNAs (38). The presence of additional reads in the latter libraries could result in nonspecific alignment to miRNA loci. For example, miR-155-3p, which is believed to represent a host miRNA passenger stand, was reported to increase upon SARS-CoV and SARS-CoV-2 infection (34), but we were unable to detect this miRNA either by sequencing or by Northern blotting. Similarly, miR-4485 was described to be up-regulated in another study (35), but it did not pass the abundance threshold (>1 counts per million [CPM]) in our smRNA-seq analysis. Also, other studies assessing the impact of various coronaviruses on host miRNA levels do not support the notion that coronaviruses have major impact on host miRNA populations (39–42). We cannot rigorously exclude the possibility that some host miRNAs levels are altered during SARS-CoV-2 infection, but if so these changes are likely minor.

Interestingly, in samples from individuals infected with SARS-CoV-2 all assessed host miRNAs seemed to be stabilized, while—similar to what is known about host mRNAs in infected cells (17)—U6B small nuclear RNA and U44 small nucleolar RNA levels decreased (*SI Appendix*, Fig. S1*H*). These results suggest that miRNAs might be resistant to the viral host-shutoff effect. Indeed, miRNAs can be very stable because of their association with Ago proteins, especially when secreted in exosomes (2, 18, 19). It is tempting to speculate that, because of their high stability, small RNAs could be successfully used as therapeutic agents against SARS-CoV-2. Regardless, we caution against examining miRNA profiles during viral infection using RT-qPCR and normalizing to ncRNAs from different classes, such as small nuclear RNAs and small nucleolar RNAs.

In this study, we discovered that SARS-CoV-2 expresses an miRNA-like small RNA, which we call CoV2-miR-O7a (Fig. 2). Of note, Singh et al. have independently reported the existence of CoV2-miR-O7a (42). Interestingly, this small RNA was also detected by Meng et al. but was not selected for further validation (12). These independent studies indicate that CoV2-miR-O7a is a functional svRNA. There are multiple examples of svRNAs expressed by RNA viruses, such as influenza (43), enterovirus 71 (44), hepatitis C (45), hepatitis A (46), polio (45), Dengue (45, 47), vesicular stomatitis (45), West Nile (45, 48), coronaviruses (10–12), and retroviruses (49, 50). It has been suggested that some svRNAs function through the host-cell RNAi pathway (12, 45, 46), but most proposed roles are independent of this machinery (10, 11, 43, 44, 51). In this study, we investigated the RNAi-related function of CoV2-miR-O7a.

CoV2-miR-O7a is derived from the beginning of the coding sequence of the ORF7a transcript (Fig. 2*B*), which is the most abundant subgenomic viral RNA detected during SARS-CoV-2 infection (34, 52). The sequence of CoV2-miR-O7a is preserved in all SARS-CoV-2 isolates and variants of concern and is related to those of two other coronaviruses (*SI Appendix*, Fig. S2). Interestingly, ORF7a deletions, which lead to decreased innate immune suppression, occur frequently in SARS-CoV-2 but affect almost exclusively the C terminus of the protein, while the N terminus—where the CoV2-miR-O7a sequence is located—is preserved (53). These data and the observed conservation of the hairpin containing CoV2-miR-O7a (22) support the notion that this sequence is functional.

The abundance of CoV2-miR-O7a ranges from low to moderate as compared to host miRNAs (Fig. 3*A*), similar to other svRNAs (10, 46, 51), and is consistent with the idea that its functionality relies on high complementarity to target mRNA, likely inducing mRNA cleavage. Indeed, we have shown that CoV2-miR-O7a associates with human Ago proteins and that it can repress human targets (Fig. 3). Using bioinformatics, we identified two host transcripts that are significantly down-regulated in the presence of a synthetic CoV2-miR-O7a, BATF2 and HSPG2 (Fig. 3 and Table 1). In addition to these, there are likely more targets that may be identified by cross-linking ligation and sequencing of hybrid [CLASH (54)] methodology. To address the functionality of CoV2-miR-O7a in the context of viral infection, we attempted to use luciferase reporters, but due to the host-shutoff effect there was almost no luciferase signal in infected cells. We avoided using antisense oligonucleotides to block CoV2-miR-O7a because they would undoubtedly also suppress production of the ORF7a protein and inhibit viral replication. Upcoming studies will focus on the construction of mutant viruses in which the ORF7a coding sequence and hairpin formation are preserved.

Another intriguing possibility is that SARS-CoV-2 uses CoV2-miR-O7a to regulate the level of its own negative-sense subgenomic RNAs, or of antigenomic RNAs (55). It is also plausible that the viral hairpin, in addition to being the source of a viral miRNA, could have an additional function independent of the RNAi pathway. RNA viruses have been shown to express regulatory svRNAs. For example, svRNA1 from enterovirus-71 binds to a viral internal ribosome entry site to regulate translation of viral proteins (44). Another example is svRNAs from influenza that associate with viral RdRp, possibly enabling the switch from transcription to replication (51).

Finally, we addressed CoV2-miR-O7a biogenesis and were able to demonstrate that its processing occurs via cellular machinery and relies on the formation of an RNA hairpin but is independent of Drosha protein (Fig. 4). Yet, it is possible that viral genes enhance this processing pathway. Many viruses bypass Drosha to produce their pre-miRNAs, e.g., by utilizing RNase Z (56, 57), Integrator complex (58), or a viral protein (such as HIV-1 TAT) (59). As shown by Singh et al. (13), Dicer, which is responsible for production of the majority of host and viral miRNAs (2, 46, 59, 60), is involved in CoV2-miR-O7a biogenesis. Future efforts will aim to uncover how the pre-CoV2-miR-O7a hairpin is cleaved out from viral transcripts.

In summary, viruses develop multiple ways to suppress host gene expression, and various overlapping mechanisms often evolve. SARS-CoV-2 regulates host mRNA expression on many levels: stability (16), export (17, 61), translation (62–64), and splicing (62). It is not surprising that yet another strategy, which utilizes the RNAi pathway, to selectively target host—and perhaps viral—transcripts could have evolved.

## Materials and Methods

### Cells.

Calu-3 (ATCC) cells were cultured in Eagle’s minimal essential medium (EMEM; ATCC) with 10% fetal bovine serum (FBS) and Penicillin/Streptomycin (Pen/Strep; GIBCO). PC-9 cells (kind gift from Craig Wilen, Yale University, New Haven, CT) were cultured in RPMI medium (Gibco) with 10% FBS and Pen/Strep. A549 (ATCC) cells were transduced as described previously (28) with hACE2 plasmid (kind gift from Benjamin Goldman-Israelow, Yale University, New Haven, CT) and cultured in F-12 medium (Gibco) with 10% FBS, Pen/Strep, and 1 μg/mL of puromycin (Gibco). Vero-E6 (ATCC), Huh7.5, and Huh7.5 Drosha knockout [kind gift from Charles Rice, Rockefeller University, New York, NY (33)] cells were cultured in Dulbecco’s modified Eagle’s medium (DMEM) with 10% FBS and Pen/Strep. Calu-3 cells were transduced either with FLAG-HA-Ago2 (28) or pLVX (for empty vector control) and cultured in the presence of 1 μg/mL of puromycin.

### Viral Stocks.

To generate SARS-CoV-2 stocks, Vero-E6 were inoculated with P2 of SARS-CoV-2 isolate USA-WA1/2020 (kind gift from Craig Wilen, Yale University, New Haven, CT) at 0.01 MOI for 1 h, after which the inoculum was replaced with DMEM with 5% FBS. After 3 d the supernatant was harvested and clarified by centrifugation, concentrated on Ultra-15 Centrifugal Filters (Amicon), aliquoted, and stored at −80 °C. Virus titers were determined by plaque assay using Vero-E6 cells. All infectious growth was performed in a Biosafety Level (BSL) 3 laboratory and was approved by the Yale University Biosafety Committee.

### Plaque Assays.

Vero-E6 cells were seeded at 4 × 10^5^ cells/well in 12-well plates. The next day, medium was removed, and cells were incubated for 1 h at 37 °C with 100 μL serially diluted sample; plates were rocked every 15 min. Next, 1 mL of the overlay media (DMEM, 2% FBS, and 0.6% Avicel) was added to each well. Three days later, the plates were fixed with 10% formaldehyde for 30 min, stained with crystal violet solution (0.5% crystal violet in 20% methanol) for 30 min, and then rinsed with water to visualize plaques.

### Samples for Small RNA Sequencing.

For small RNA sequencing, 5 × 10^5^ of Calu-3, 5 × 10^5^ of PC-9, or 1 × 10^5^ of A549-hACE2 cells were plated per well in six-well plates. Cells were infected with SARS-CoV-2 at MOI of either 5 or 0.05 for 1 h in 200 µL of inoculum and incubated for 1 h at 37 °C. After that, the cells were rinsed with phosphate-buffered saline to remove the unbound virus and fresh media were added. Cells were incubated for either 6 h and 24 h (MOI 5) or 48 h (MOI 0.05). In addition, mock-treated controls were collected after 24 h. Cells were harvested by removing the media and adding TRIzol at each time point; supernatants were kept to assess viral titer by plaque assay. RNA isolation and library preparation was performed under BSL2+ confinement; RNA concentration was measured by Qubit Fluorimeter (Thermo Fisher) inside a biosafety cabinet. One microgram of total RNA was used for library preparation using NEXTflex v2 kit (PerkinElmer) according to the manufacturer’s instructions. Libraries were amplified for 16 cycles and the complementary DNA (cDNA) libraries were sequenced on the HiSeq 2500 Illumina platform.

### Small RNA Data Processing.

The reads were trimmed of adaptors using Cutadapt (65) with the following settings: -u 4 -O 7 -a N{4}TGGAATTCTCGGGTGCCAAGG -q 10 -m 18 -M. The reads were mapped with bowtie2 (66) (–very-sensitive-local) to an index containing human and SARS-CoV-2 genomes. miRNAs were counted by using featureCounts (67) and annotations obtained from miRBase (68). For Ago immunoprecipitation (IP) counts, CoV2-miR-O7a was added to the annotation file and treated as a host miRNA. Differential expression was determined using edgeR (69). Track visualization was performed using an IGV browser (70) of generated with BEDtools (71) bed files.

The initial experiments were done in two biological replicates at high MOIs (series A and B); subsequently, the experiment was repeated for the third time, also adding a low MOI condition (series 1, 2, and 3). Because the series A and B and 1 through 3 were sequenced separately, for differential expression analysis, we used mock samples from series A, B, 1 and 2; all other conditions had three replicates.

### Patient Samples and RT-qPCR.

The human study reported here from which nasopharyngeal swab samples were obtained was approved by the Yale Human Research Protection Program (Protocol 2000027971). Subjects from whom nasopharygeal swabs were collected were enrolled in a randomized, placebo-controlled, double-blind clinical trial for early treatment of SARS-CoV-2 infection (https://clinicaltrials.gov/, NCT04353284). Each provided written informed consent both for enrollment in the trial and for additional uses of the samples including work described herein.

The nasopharyngeal swab samples were extracted using MagMAX mirVana Total RNA Isolation Kit (catalog no. A27828), with the script A27828_FLEX_Biofluids for miRNA extraction from biofluid samples on the KingFisher FLEX-96 Magnetic Particle Processor. Briefly, each 100-μL specimen of nasopharyngeal swab in transportation medium was processed and the miRNA-enriched RNA was collected in 50 μL of elution buffer. miRNA RT-qPCR was performed using TaqMan MicroRNA Assay (Applied Biosystems); for miR-16, miR-210-3p, miR-31-3p, miR-193-5p, miR-193-3p, U6B, and U44 predesigned assays were obtained (catalog no. 4427975), and for miR-O7a a custom assay to detect the sequence UUCUUGGCACUGAUAACAC was ordered (catalog no. 4398987). RT-qPCR was performed according to the manufacturer’s guidelines; briefly, cDNA was prepared separately for each miRNA using the TaqMan MicroRNA Reverse Transcription Kit (Applied Biosystems) and qPCR was performed using the TaqMan Fast Advanced Master Mix (Applied Biosystems). For detection of viral RdRp, cDNA was prepared using random primers and SuperScript III (Invitrogen) and qPCR was performed according to the protocol (72) using oligonucleotides and a probe ordered from IDT (sequences are given in *SI Appendix*, Table S2). All work was done in BSL2+ conditions.

### Northern Blot Analysis.

Calu-3 cells (5 × 10^6^) were infected with SARS-CoV-2 at MOI 0.05 in T75 flasks for 1 h, after which the inoculum was replaced by fresh media, and cells were incubated for either 24 h or 48 h (mock-treated samples were collected after 48 h). Samples were inactivated for 30 min in 5 mL of TRIzol and RNA was isolated in BSL2+ conditions. Ten to 15 µg of total RNA was separated by 15% urea-PAGE, electrotransferred to Hybond-NX membrane (Amersham), and cross-linked with 1-ethyl-3-(3-dimethylaminopropyl) carbodiimide (EDC) (73). miRNAs were detected using ^32^P 5′-radiolabeled DNA probes. Densitometry was performed by using Quantity One.

### miR-210-3p Target and Reanalysis of Public Data.

Counts from RNA-seq experiments of lung biopsies were obtained from refs. 15 and 16. The data were divided into miR-210-3p targets [data from miRTarBase (74), excluding “weak” targets] and other transcripts. The data were plotted as cumulative distribution functions and compared to each other using Wilcoxon test.

### IP.

For anti-pan Ago IP, 5 × 10^5^ Calu-3 cells or 1 × 10^5^ A549-hACE2 cells were infected with SARS-CoV-2 at MOI 5 for 24 h. Cells were detached with trypsin-EDTA (ethylenediaminetetraacetic acid), pelleted, and resuspended in 500 µL of NET-2 buffer (50 mM Tris [pH 7.5], 150 mM NaCl, and 0.05% Nonidet P-40). After 30 min of cell lysis inside the biosafety cabinet (BSC), the nuclei were pelleted and supernatants were transferred to 2.0-mL screw-top tubes with O-rings containing magnetic beads (SureBeads Protein G Magnetic Beads; NEB) coupled to antibodies (anti-pan Ago antibody, clone 2A8; Sigma Millipore). Ten percent of supernatants were kept for input (in TRIzol). The tubes were sealed, decontaminated, and placed inside 15-mL screw-top falcon tubes. The 15-mL falcon tubes were decontaminated and transferred to the refrigerator containing the rotator where immunoprecipitation took place. After 6 h, the beads were washed inside BSC seven times with NET-2 by gentle pipetting and with using a magnetic stand. Finally, the magnetic beads were resuspended in 1 mL of TRIzol. Described procedures were performed in a BSL 3 laboratory and were approved by the Yale University Biosafety Committee. RNA isolation and library preparation (as described above) was performed under BSL2+ confinement.

For anti-HA IP, 5 × 10^6^ of Calu-3 cells, transduced with either EV or Ago2, were infected with SARS-CoV-2 at MOI 0.1 for 48 h. Cells were detached with trypsin-EDTA, pelleted, and resuspended in 2 mL of Pierce IP Lysis Buffer (Thermo Fisher). Cells were incubated at room temperature for 30 min (approved by the Yale University Biosafety Committee SARS-CoV-2 inactivation method). After this time, the samples were moved to the BSL2+ laboratory, sonicated using a Diagenode Bioruptor Pico sonication device and IP was performed using anti-HA Magnetic Beads (Pierce). Beads were resuspended in TRIzol and RNA was extracted and analyzed by Northern blot as described above.

### Western Blot Analysis.

IPs were done as described above (excluding SARS-CoV-2 infection), and supernatants were mixed with 4× sodium dodecyl sulfate polyacrylamide gel electrophoresis (SDS-PAGE) loading buffer. Typically, 25 µL (corresponding to ∼40 µg total protein) were separated on a 10% SDS-PAGE gel and electrotransferred to a poly(vinylidene difluoride) membrane (Bio-Rad). After blocking with 5% milk in 1× TBST (20 mM Tris [pH 7.5], 150 mM NaCl, and 0.1% Tween 20), the membrane was probed with the appropriate antibodies and detected with Western Lightning Plus-ECL (PerkinElmer) using a Gbox (Syngene). Primary antibodies used were anti-FLAG M2 (Sigma Millipore), anti-Ago2 (MA5-23515; Invitrogen), and anti-GAPDH (Cell Signaling).

### Target Predictions.

Custom Perl scripts that use the RNAduplex algorithm—part of the Vienna RNA Package (53)—were used to hybridize the CoV2-miR-O7a sequence to mRNA transcripts obtained from GENCODE (v38) (75) fragmented into 50-nt windows with 5-bp steps. Fragments with the lowest hybridization energy (ΔG) were chosen for further analysis.

### Synthetic RNA Transfections and qPCR.

Synthetic RNAs (see *SI Appendix*, Table S2 for sequences) were annealed by heating equimolar concentrations for 1 min at 90 °C in siRNA buffer (Horizon, 60 mM KCl, 6 mM Hepes [ pH 7.5], and 0.2 mM MgCl_2_) and then incubating for 1 h at 37 °C. HEK293T cells (5 × 10^5^) were transfected with 30 μM of either CoV2-miR-O7a or control siRNA by using Lipofectamine RNAiMAX Transfection Reagent (Invitrogen). Cells were collected 48 h later, RNA extractions were performed using TRIzol, and samples were treated with RQ1 DNase (Promega). cDNA was made using SuperScript III and random primers (Invitrogen), and qPCR was performed using FastStart Essential DNA Green Master (Roche). Primer sequences are given in *SI Appendix*, Table S2. Results were analyzed using the comparative Ct method (76).

### In Vitro RNA Transcription and Processing.

The sequences were PCR-amplified from templates containing desired mutations by using flanking primers; the forward primer contained the T7 promoter (see *SI Appendix*, Table S2 for sequences). The PCR product was purified and used for in vitro transcription at a final concentration 25 ng/µL. The reaction was carried out for 4 h at 37 °C and contained 400 mM Hepes [pH 7.5], 120 mM MgCl_2_, 200 mM dithiothreitol, 10 mM spermidine, 4 mM of each ribonucleoside triphosphate , 20 U RNase Inhibitor (Roche), and 5 U of laboratory-made T7 RNA polymerase. The product was purified by 8 M urea 6% PAGE, extracted in G-50 buffer (20 mM Tris [pH 7.5], 0.3 M NaOAc, 2 mM EDTA, and 0.1% SDS), and phenol-extracted. To ensure hairpin formation, RNAs were annealed by heating in siRNA buffer for 1 min at 90 °C and then incubating for 1 h at 37 °C.

HEK293Ts, Huh7.5, or Huh7.5 knockout (KO) cells (5 × 10^5^) were transfected with 30 μM of in vitro-transcribed RNAs by using Lipofectamine RNAiMAX Transfection Reagent (Invitrogen). Cells were collected 6 h later, RNA extractions were performed using TRIzol, and samples were processed for Northern blotting as described above.

### Luciferase Reporter Assays.

Four sites complementary to CoV2-miR-O7a (or scrambled sequence as control) separated by 20-nt-long spacer sequences were PCR-amplified using four primers overlapping at the unique spacer sequences (listed in *SI Appendix*, Table S2). The PCR products were cloned downstream of *Renilla* luciferase of psiCHECK(TM)-2 vector (Promega) using XhoI and NotI sites.

HEK293Ts, Huh7.5, or Huh7.5 KO cells (5 × 10^5^) were transfected with 30 μM of in vitro-transcribed RNAs by using Lipofectamine RNAiMAX Transfection Reagent (Invitrogen). Cells were collected 6 h later, RNA extractions were performed using TRIzol, and samples were processed for Northern blotting as described above. Twenty-four hours later, 10 ng psiCHECK reporters and 2 μg pBlueScript II (Stratagene) were transfected using TransIT-293 Transfection Reagent (Mirus). After an additional 24 h, Firefly and *Renilla* luciferase activities were measured by the Dual-Luciferase Reporter Assay System (Promega) on a GloMax-Multi+ Microplate Multimode Reader (Promega) according to the manufacturer’s instructions.

## Supplementary Material

Supplementary File

## Data Availability

The RNA sequencing data generated in this study have been deposited in the National Center for Biotechnology Information Gene Expression Omnibus (GEO) database (accession no. GSE183280). All other data are included in the article and/or supporting information.Custom Perl scripts (available in the *SI Appendix*) that use the RNAduplex algorithm—part of the Vienna RNA Package part of the Vienna RNA Package (53)—were used to hybridize the CoV2-miR-O7a sequence to mRNA transcripts obtained from GENCODE (v38) (75) fragmented into 50-nt windows with 5-bp steps.

## Change History

January 21, 2022: The byline has been updated.
