# Supplementary material for: SARS-CoV-2 expresses a microRNA-like small RNA able to selectively repress host genes
Source: Proc Natl Acad Sci U S A. 2021 Dec 13;118(52):e2116668118. doi: 10.1073/pnas.2116668118 (PMC8719879; doi:10.1073/pnas.2116668118)
Supplement: Supplementary File [file pnas.2116668118.sapp.pdf]

Supplementary figures

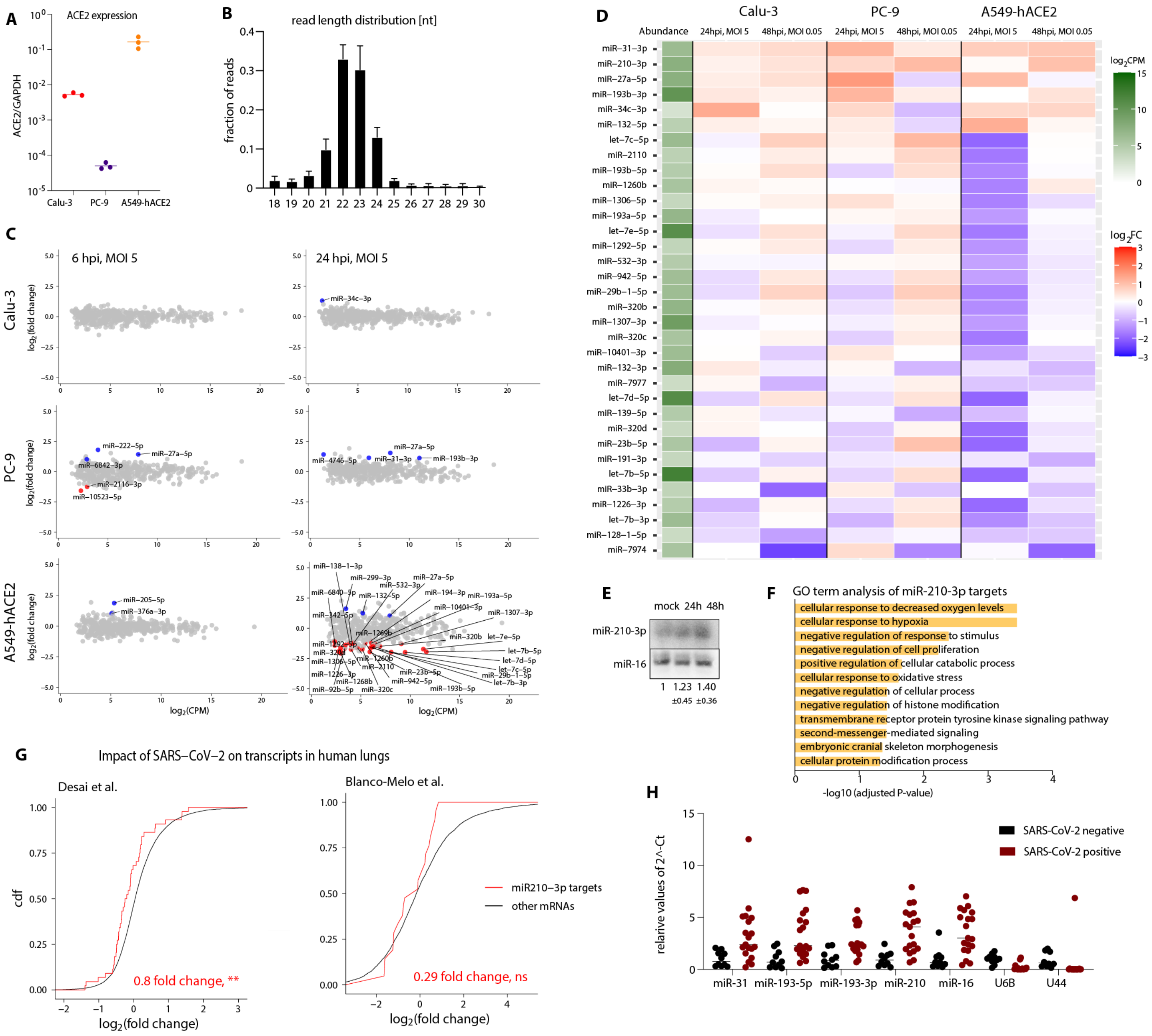

Figure S1. SARS-CoV-2 infection has minimal impact on host miRNA levels. A) Relative transcript levels of ACE2 in the three lung cell lines used in the study. **B)** A bar graph summarizing the sizes of obtained reads from all conditions sequenced. Error bars signify SD. **C)** Plots summarizing the fold-change in miRNA levels upon SARS-CoV-2 infection of various cell lines, n = 3. Colored dots denote miRNAs that significantly ($p < 0.05$) changed (at least two-

fold). Significance was calculated using edgeR. **D)** A heat-map showing fold changes (FC) in miRNA levels after SARS-CoV-2 infection. The data are shown only for miRNAs that significantly changed (at least 2-fold) in any of the conditions. **E)** Northern blot showing that miR-210-3p slightly increases after infection with SARS-CoV-2 infection of Calu-3 cells at MOI 0.05, n = 5. **F)** GO term analyses for miR-210-3p targets suggest that this miRNA regulates responses to various stimuli. ** - p = 0.004, as calculated by Wilcoxon test. **G)** miR-210-3p targets (13) exhibit stronger downregulation than other host mRNAs after SARS-CoV-2 infection in human lungs (data replotted from (14, 15)). **H)** Host miRNAs may escape host shutoff induced by SARS-CoV-2 infection. hpi — hours post infection; MOI — multiplicity of infection; CPM — counts per million; cdf — cumulative distribution function.

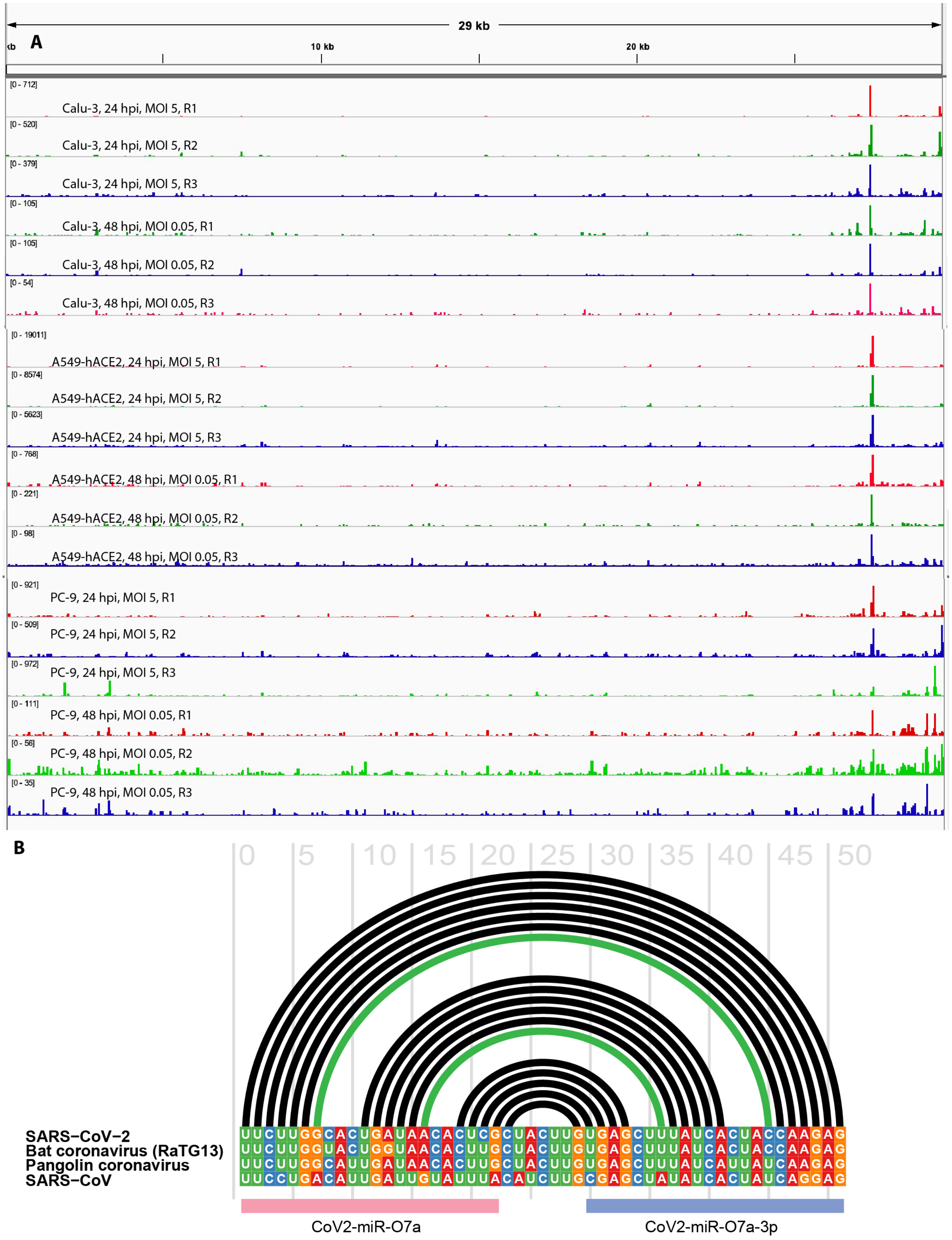
A
29 kb
10 kb
20 kb
[0 - 712]
Calu-3, 24 hpi, MOI 5, R1
[0 - 520]
Calu-3, 24 hpi, MOI 5, R2
[0 - 379]
Calu-3, 24 hpi, MOI 5, R3
[0 - 105]
Calu-3, 48 hpi, MOI 0.05, R1
[0 - 105]
Calu-3, 48 hpi, MOI 0.05, R2
[0 - 54]
Calu-3, 48 hpi, MOI 0.05, R3
[0 - 19011]
A549-hACE2, 24 hpi, MOI 5, R1
[0 - 8574]
A549-hACE2, 24 hpi, MOI 5, R2
[0 - 5623]
A549-hACE2, 24 hpi, MOI 5, R3
[0 - 768]
A549-hACE2, 48 hpi, MOI 0.05, R1
[0 - 221]
A549-hACE2, 48 hpi, MOI 0.05, R2
[0 - 98]
A549-hACE2, 48 hpi, MOI 0.05, R3
[0 - 921]
PC-9, 24 hpi, MOI 5, R1
[0 - 509]
PC-9, 24 hpi, MOI 5, R2
[0 - 972]
PC-9, 24 hpi, MOI 5, R3
[0 - 111]
PC-9, 48 hpi, MOI 0.05, R1
[0 - 56]
PC-9, 48 hpi, MOI 0.05, R2
[0 - 35]
PC-9, 48 hpi, MOI 0.05, R3
B
0 5 10 15 20 25 30 35 40 45 50
SARS−CoV−2 UUCUUGGCACUGAUAACACUCGCUACUUGUGAGCUUUAUCACUACCAAGAG
Bat coronavirus (RaTG13) UUCUUGGUACUGGUAACACUUGCUACUUGUGAGCUUUAUCACUACCAAGAG
Pangolin coronavirus UUCUUGGCAUUGAUAACACUUGCUACUUGUGAGCUUUAUCAUUAUCAAGAG
SARS−CoV UUCCUGACAUUGAUUGUAUUUACAUCUUGCGAGCUAUAUCACUAUCAGGAG
CoV2-miR-O7a
CoV2-miR-O7a-3p

Figure S2. SARS-CoV-2 expresses a small RNA derived from the ORF7a sequence. **A)** Sequencing tracks for all replicates are shown. **B)** Location of CoV2-miR-O7a and CoV2-miR-O7a-3p annotated on an alignment of coronavirus reference sequences. The hairpin base pairs are indicated by arcs above the sequence alignment with the two previously-identified covarying base pairs (21) indicated in green. Alignment and base pairs visualized using the program R-CHI (78).

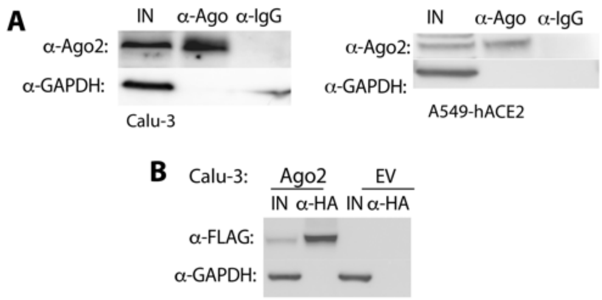

Figure S3. Western blots confirming anti-Argonaute immunoprecipitation. A) anti-pan Ago IPs detected with anti-Ago2 antibodies. **B)** anti-HA IP of FLAG-HA-tagged Ago2 detected with anti-FLAG antibodies.

miR-16 probe	CGCCAATATTTACGTGCTGCTA
miR-210-3p probe	TCAGCCGCTGTCACACGCACAG
CoV2-miR-O7a probe	CGAGTGTTATCAGTGCCAAGAA
CoV2-miR-O7a-3p probe	TCTTGGTAGTGATAAAGCTCAC
RdRp_SARSr-F (qPCR primer)	GTGARATGGTCATGTGTGGCGG
RdRp_SARSr-R (qPCR primer)	CARATGTTAAASACACTATTAGCATA
RdRp_SARSr-P2 (qPCR probe)	FAM-CAGGTGGAACCTCATCAGGAGATGC-BBQ
Synthetic CoV2-miR-O7a	/5Phos/rUrUrCrUrUrGrGrCrArCrUrGrArUrArArCrArCrUrC
Passenger strand for CoV2-miR-O7a	/5Phos/rGrUrGrUrUrArUrCrArCrUrGrCrCrUrArGrArUrUrG
siContol	rArArGrCrGrArUrArCrCrUrCrGrUrGrUrGrUrGrArUrU
T7_F (amplification)	GAATTTAATACGACTCACTATAGGTTCTCCTCTAAACGAACATGAA
T7_R (amplification)	GAATGTACCTCTAACACACTCTTGGT

WT (template)	GGTTCTCCTCTAAACGAACATGAAAATTATTCTTTTCTTGGCACTGATAA CACTCGCTACTTGTGAGCTTTATCACTACCAAGAGTGTGTTAGAGGTACA
m3p (template)	GGTTCTCCTCTAAACGAACATGAAAATTATTCTTTTCTTGGCACTGATAA CACTCGCTACTTCTCTCCTTATAGACTACCAAGAGTGTGTTAGAGGTACA
m5p (template)	GGTTCTCCTCTAAACGAACATGAAAATTATTCTTTAGAACGCACTCTATT CACTCGCTACTTGTGAGCTTTATCACTACCAAGAGTGTGTTAGAGGTACA
ACE2_F	CAAGAGCAAACGGTTGAACAC
ACE2_R	CCAGAGCCTCTCATTGTAGTCT
HSPG2_F	TTGACCAACCCGATGACTTCA
HSPG2_R	CGCCATAGGAGTCCACCTT
BATF2_F	TCAGGAAGCAGCCTTAGCAC
BATF2_R	AGGAGAAGGAGGAGCAGAGG
ZBTB5_F	CCATTTCAGATGTTACACCGGA
ZBTB5_R	TCGCACTATCTTCCTGGTTATCA
BATF1_F	GAGAACTGCGCTTACTAGGCT
BATF1_R	GCCTCAAGTGGCATGAGTCT
PRKCH_F	CGGGATCAACATCCCACACA
PRKCH_R	ACCGCATTTACCCCACAGTT
FAM13A_F	TGCTCATGTACCCCAAGTCAG
FAM13A_R	CTTGTCTCTCCCATGTCGAAC
SUFU_F	GCCTGAGTGATCTCTATGGTGA
SUFU_R	TCTCTCTTCAGACGAAAGGTCAA

Table 2. Oligonucleotides used in the study
